# **Oral Diagnosis of Lepromatous Leprosy in a Hyperendemic Region of Brazil**

**DOI:** 10.1007/s12105-026-01945-9

**Published:** 2026-07-02

**Authors:** Ana Cláudia Garcia Rosa, Marcos Phelipe Araujo Andrade Alves, Regina Garcia Dorta

**Affiliations:** 1 Federal University of Tocantins, School of Medicine, Palmas, Tocantins Brazil; 2 Afya Faculty of Medical Sciences, School of Dentistry, Palmas, Tocantins Brazil; 3 Maurício de Nassau University Center, School of Dentistry, Palmas, Tocantins Brazil; 4 São Leopoldo Mandic School of Dentistry, Campinas, São Paulo Brazil

**Keywords:** Leprosy, Hansen`s disease, Oral manifestations, Lepromatous leprosy, Histopathology, Oral biopsy

## Abstract

**Background:**

Leprosy is a chronic infectious disease caused by Mycobacterium leprae that primarily affects the skin and peripheral nerves. Oral manifestations are rare and typically occur in advanced cases of multibacillary lepromatous leprosy.

**Case Presentation:**

An 18-year-old male from Tocantins, a hyperendemic region of Brazil, presented with widespread mucocutaneous papulonodular lesions affecting the face and extremities, as well as nodular and ulcerative lesions of the hard and soft palates and the uvula. Histopathological analysis revealed a diffuse subepithelial inflammatory infiltrate predominantly consisting of foamy macrophages exhibiting Virchow-like characteristics. Immunohistochemistry showed diffuse CD68 positivity. Fite-Faraco and Ziehl-Neelsen staining revealed numerous intracytoplasmic acid-fast bacilli. These findings, together with clinical features, established the diagnosis of lepromatous leprosy.

**Conclusion:**

Although oral manifestations of leprosy are uncommon, they may represent important diagnostic indicators of advanced multibacillary disease. Oral biopsy, combined with histopathological examination and special stains, remains essential for definitive diagnosis, particularly in hyperendemic regions where other granulomatous infectious diseases may coexist.

## Case Report

Leprosy is a chronic infectious disease caused by *Mycobacterium leprae*, affecting the skin and peripheral nerves. Oral manifestations are typically associated with advanced multibacillary disease and may facilitate diagnosis in hyperendemic regions, where delayed recognition remains common [1, 2].

An 18-year-old male from Tocantins, Brazil, a hyperendemic region [3], was referred for evaluation of a palatal lesion noted three months earlier. The patient reported recurrent swelling and prolonged bleeding after minor trauma, but denied pain or sensory impairment. No relevant medical history or medication use was reported.

**Fig. 1:**
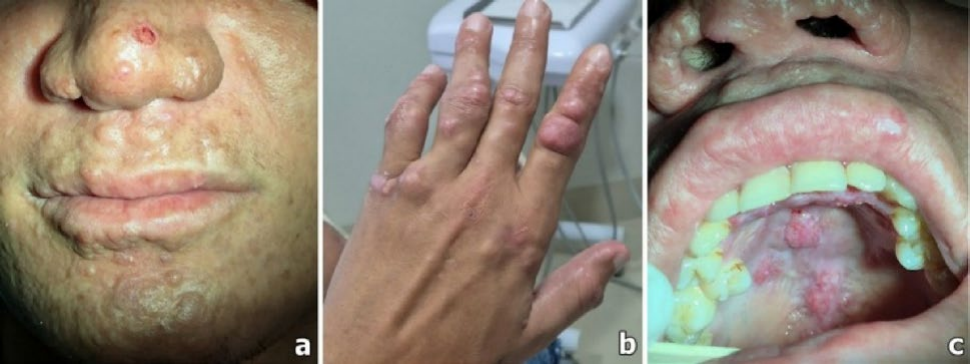
Clinical manifestations of multibacillary lepromatous leprosy. **a** Extraoral view showing diffuse papulonodular infiltrative lesions involving the nasal dorsum, nasal alae, upper lip, perioral region, and chin, with focal ulceration and crusting of the nasal lesion. **b** Multiple nodular lesions affecting the dorsal surfaces of the fingers and periarticular regions. **c** Intraoral view demonstrating multiple nodular and ulcerative lesions involving the hard palate, predominantly along the midline

Extraoral examination revealed diffuse papulonodular infiltrative lesions involving the nasal dorsum, nasal alae, upper lip, perioral region, and chin. The lesions exhibited smooth, shiny surfaces ranging from skin-colored to erythematous, with focal ulceration and central crusting. Nasal enlargement and distortion of the alar contour were observed (Fig. 1a). Multiple nodular lesions were also present on the dorsal surfaces of the fingers and periarticular regions, some showing crusting and digital deformity (Fig. 1b). Intraoral examination demonstrated multiple papular and nodular lesions on the hard palate, predominantly along the midline, with erythematous surfaces and focal superficial ulceration. Other smaller nodular lesions were observed in the adjacent palatal mucosa (Fig. 1c).

The disseminated mucocutaneous presentation suggested an infectious or granulomatous disease. Differential diagnoses included leprosy, mucocutaneous leishmaniasis, tuberculosis, and paracoccidioidomycosis. Laboratory findings were unremarkable. An incisional biopsy of the palatal lesion was performed.

**Fig. 2:**
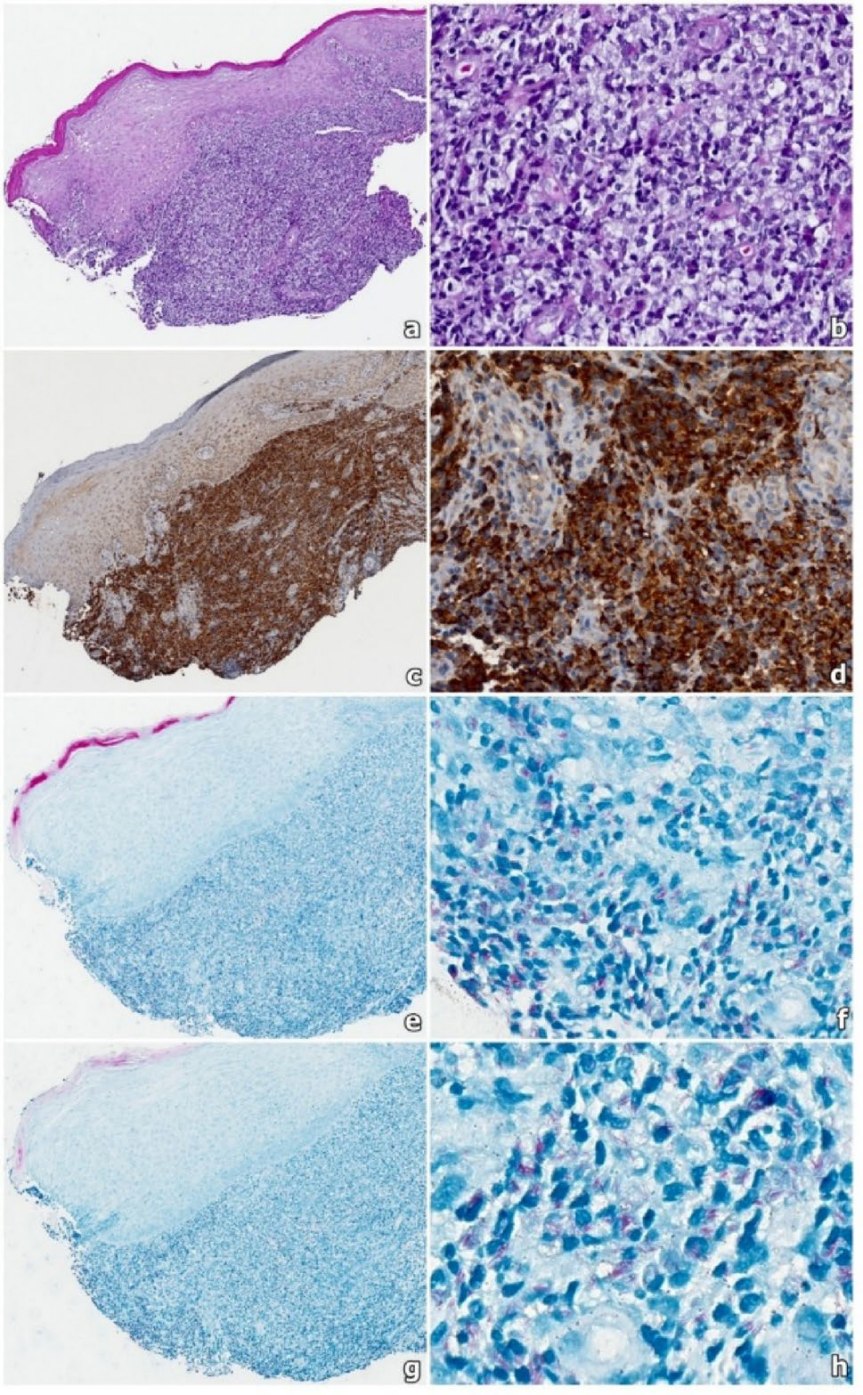
Histopathological, immunohistochemical, and histochemical features characteristic of oral lepromatous leprosy. (**a**, **c**,**e**, **g**) Low-power views and (**b**, **d**, **f**, **h**) corresponding high-power views. (**a**, **b**) H&E-stained sections demonstrating stratified squamous epithelium overlying a dense, diffuse subepithelial inflammatory infiltrate composed predominantly of foamy macrophages with Virchow-like features. (**c**, **d**) CD68 immunohistochemical staining showing diffuse positivity within the histiocytic/macrophagic infiltrate. (**e**, **f**) Fite-Faraco stain revealing numerous intracytoplasmic acid-fast bacilli within foamy macrophages. (**g**, **h**) Ziehl-Neelsen stain confirming abundant acid-fast bacilli within the inflammatory infiltrate.

Microscopic examination revealed stratified squamous epithelium overlying a dense diffuse subepithelial inflammatory infiltrate predominantly composed of vacuolated foamy macrophages intermixed with scattered lymphocytes. The foamy histiocytic cells exhibited abundant pale eosinophilic to clear cytoplasm, consistent with Virchow cells. A focal subtle grenz zone was identified, and well-formed epithelioid granulomas were absent (Fig. 2a and b). Immunohistochemical analysis demonstrated diffuse strong CD68 positivity, confirming the predominance of histiocytic cells (Fig. 2c and d). Special stains, including Fite-Faraco (Fig. 2e and f) and Ziehl-Neelsen (Fig. 2g and h), revealed numerous intracytoplasmic acid-fast bacilli within foamy histiocytes. Correlation of the clinical, histopathological, immunohistochemical, and histochemical findings established the diagnosis of lepromatous leprosy according to the Ridley-Jopling classification [4].

At follow-up three months after biopsy, progression of the oral lesions was observed, including enlargement of the palatal lesion and the development of multiple nodular lesions involving the hard and soft palate and the uvula. Progression of the nasal dorsum and hand lesions was also noted. The patient was referred for multidrug therapy (MDT) with rifampicin, clofazimine, and dapsone according to World Health Organization recommendations [5] and is currently undergoing treatment.

Although oral manifestations of leprosy are uncommon, they may represent important diagnostic indicators of advanced multibacillary disease. Oral biopsy, combined with histopathological examination and special stains, remains essential for definitive diagnosis. Failure to recognize these manifestations in young individuals may contribute to delayed diagnosis and continued disease transmission. This is particularly relevant in hyperendemic regions, where delayed diagnosis is common, and other granulomatous infectious diseases may coexist. Increasing global migration also reinforces the importance of recognizing leprosy in non-endemic regions, where imported cases, although uncommon, may still occur.

## Data Availability

No datasets were generated or analysed during the current study.
